# Predicting the Distribution of *Neoceratitis asiatica* (Diptera: Tephritidae), a Primary Pest of Goji Berry in China, under Climate Change

**DOI:** 10.3390/insects15080558

**Published:** 2024-07-23

**Authors:** Zhongkang Song, Guanghui Fan, Changrong Deng, Guozhen Duan, Jianling Li

**Affiliations:** Qinghai Plateau Tree Genetics and Breeding Laboratory, Laboratory for Research and Utilization of Qinghai Tibet Plateau Germplasm Resources, Academy of Agriculture and Forestry Sciences, College of Agriculture and Animal Husbandry, Qinghai University, Xining 810016, China; zkangsong@163.com (Z.S.); guanghui_fan_mail@163.com (G.F.); dengchang_rong@126.com (C.D.); 18848110959@163.com (G.D.)

**Keywords:** *Neoceratitis asiatica*, goji berry, MaxEnt, climate warming, geographical distribution

## Abstract

**Simple Summary:**

The fruit fly, *Neoceratitis asiatica* Becker, is a frugivorous pest that causes substantial losses in the production of the goji berry, *Lycium barbarum* L. Identifying its distribution is significant for the monitoring and prevention of the pest. In this study, the MaxEnt model with optimized parameters was employed to predict the current and future (2050s and 2070s) distribution of the pest. We found that temperature and precipitation were the primary environmental factors influencing its distribution. The suitable habitats were predominantly distributed in northwestern China under various climate scenarios, and the projected suitable area in the future generally exhibited a decrease compared with the current projections.

**Abstract:**

Climate warming affects the growth and development of pests, resulting in changes in their geographical distribution, which increases the difficulty in terms of prevention and control. The fruit fly, *Neoceratitis asiatica* (Becker), is a predominant frugivorous pest that causes serious yield loss in the goji berry, *Lycium barbarum* L. In recent years, with the expansion of cultivation area, the damage induced by the pest has become increasingly severe, significantly impeding the production of the goji berry. In this study, the potential suitable habitats of *N. asiatica* under current and future climate scenarios were simulated and predicted using the optimal MaxEnt model, based on the screening distribution records and environmental factors. The changes in the pest distribution under climate change were determined using ArcGIS. The results showed that the best combination of parameters for MaxEnt were feature combination (FC) = LQPT and regularization multiplier (RM) = 1. The dominant environmental factors influencing pest distribution were mean temperature of driest quarter, mean temperature of coldest quarter and precipitation of coldest quarter. Under different climate conditions, the suitable habitats of the pest primarily ranged between 27°–47° N and 73°–115° E. Under current climate conditions, the area of moderately and highly suitable habitats was 42.18 × 10^4^ km^2^, and mainly distributed in Inner Mongolia (13.68 × 10^4^ km^2^), Gansu (9.40 × 10^4^ km^2^), Ningxia (5.07 × 10^4^ km^2^), Qinghai (4.10 × 10^4^ km^2^), and Xinjiang (3.97 × 10^4^ km^2^) Provinces. Under future climate scenarios, the suitable area was projected to be lower than the current ones, except SSP245–2050s and SSP370–2070s, and the centroids of suitable habitats were mainly shifted to the northeast, except SSP370–2050s and SSP585–2070s. Our results provide valuable guidance for the monitoring and management of *N. asiatica*, as well as the selection of pest-free goji berry cultivation sites.

## 1. Introduction

Global climate warming is a result of both natural factors, mainly solar activity, and human activities, primarily greenhouse gas emissions [1], which exerts a profound impact on biodiversity and the distribution patterns of insects [2]. According to the reports of the Intergovernmental Panel on Climate Change (IPCC), the global temperature has risen by about 0.8 °C over the past 100 years and is expected to continue to increase as a result of escalating emissions of greenhouse gas [3,4]. As ectothermic animals, insect developmental duration will be shortened, and reproductive rates will be accelerated with the rise in temperature [4]. Meanwhile, the overwintering survival rate will also increase [5]. Therefore, climate warming may facilitate the spread of pests and aggravate damage [6]. The geographical ranges of pest distribution are expected to expand from lower to higher altitudes and latitudes, potentially leading to more severe agricultural production damage [7]. Therefore, identifying the impacts of climate change on pest distribution is crucial for pest prevention and control, and will provide valuable guidance for crop cultivation.

*Lycium barbarum* L., commonly known as the goji berry, is a medicinal and edible herb that is indigenous to China, renowned for its hepatoprotective and nephrotonic properties, as well as its potential in enhancing vitality and improving ocular health [8]. Due to its exceptional drought and salt stress tolerance, the goji berry exhibits high suitability for cultivation in Northwest China, specifically in Ningxia, Qinghai, Gansu, Inner Mongolia, and Xinjiang Provinces [9]. In recent years, the cultivation of this plant has gained significant prominence as a highly sought-after Chinese traditional medicinal material, with an annual consumption exceeding 300,000 tons and the cultivation area surpassing 1200 km^2^ [10]. Consequently, it has emerged as a pivotal source of income for local farmers and herdsmen in Northwest China. However, due to the simultaneous occurrence of vegetative and reproductive growth in the goji berry, along with its luxuriant foliage, it exhibits higher susceptibility to pests compared to other herbs [11]. Thus, the absence of timely prevention and control measures often results in a substantial reduction in yield. What is more, with the recent expansion of cultivation area and the ongoing warming climate, the detrimental impacts caused by pests are progressively intensifying, posing an increasingly substantial threat to the herb [12].

The fruit fly, *Neoceratitis asiatica* Becker, is a primary frugivorous pest on goji berries and is primarily distributed in Northwest China (Ningxia, Qinghai, Gansu, Inner Mongolia, and Xinjiang Provinces), Kazakhstan, and Turkmenistan [13]. These regions are situated in the hinterland of the Eurasian continent with a typical arid and semiarid climate. The annual precipitation in these regions is consistently below 400 mm, with a primary concentration in summer, while winter is characterized by arid and frigid conditions [14]. The fly deposits its eggs in immature fruits, and the emerging larvae feed and inhabit the fruits, resulting in fruit wilting and decay [15]. Due to the concealed nature of the larvae within the fruit, the control efficacy of conventional pesticides, such as Lambda-cyhalothrin and Spinetoram, is poor because of their limited systemic absorption [13]. In recent years, the incidence and damage of the pest have progressively increased with the expansion of goji berry cultivation, particularly in organic plantations where chemical pesticides are prohibited, resulting in significant economic losses to herb production [16]. It was estimated that more than 20% of the annual losses are caused by the pest [17]. Previous studies have primarily focused on biology [15,18] and management [19,20] of the pest, while the geographic ranges of this pest remain uncertain. Identifying a suitable habitat is significant for pest prevention and cultivation site selection.

Species distribution models (SDMs) are powerful tools for predicting the geography of species based on distributional coordinates and environmental factors [21]. They are widely applied in risk assessment of invasive alien species, prediction of the species’ geographical distribution, and formulation of conservation strategies for endangered species [22]. Among the available SDMs, the maximum entropy model (MaxEnt) is widely employed for its robust predictive performance, particularly in scenarios with limited distribution records and uncertain correlations among environmental factors [23,24]. The potential distribution of the goji berry [9,10] and its pests, including aphid, *Aphis* sp. [25] and gall mite, *Aceria macrodonis* Keifer [26] were extensively studied using MaxEnt, which was proven to be effective in monitoring the impact of climate change on the goji berry and its pests.

In this study, the optimal MaxEnt model was utilized to predict the current and future geographical distribution of *N. asiatica* under climate warming, as well as to identify the predominant environmental factors influencing the pest distribution. Our study will provide valuable insights for pest monitoring and management, and for identifying suitable pest-free sites for goji berry cultivation.

## 2. Materials and Methods

### 2.1. Acquisition and Processing of Distribution Records and Environmental Data

**Figure 1:**
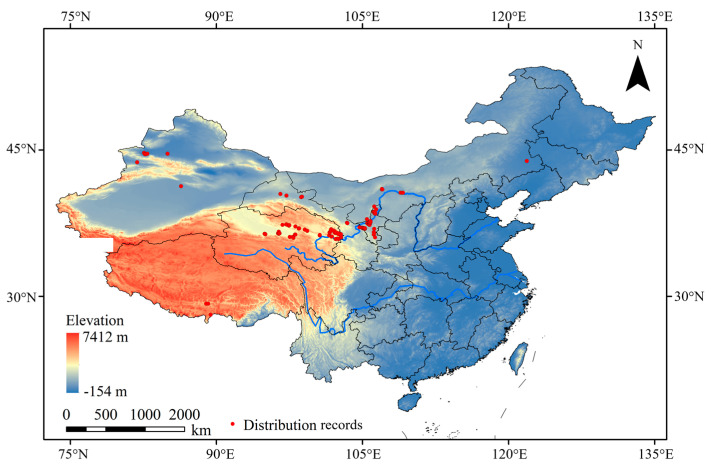
Distributions of occurrence records of *N. asiatica* in China [Inspection number: GS (2019) 1822].

In this study, a total of 166 distribution records of *N. asiatica* were collected from the following sources: (1) scientific publications [13,27]; (2) Global Biodiversity Information Facility (GBIF, https://www.gbif.org/occurrence/search?taxon_key=1628203, assessed on 14 November 2023) and Medicinal Plant Pests Database (MPPD, https://www.pests.cn/index/tu?pic_name=%E6%9E%B8%E6%9D%9E%E5%AE%9E%E8%9D%87, assessed on 14 November 2023); (3) our investigation results in 2022–2023. To reduce sampling bias, these records were filtered using the spatial analysis function of ArcGIS 10.7 (https://www.esri.com/en-us/arcgis/products/arcgis-desktop/resources, assessed on 15 November 2023). Only one record was retained if the distance between two records was less than 10 km. Ultimately, a total of 115 valid records of *N. asiatica* were obtained (Supplementary Table S1), all of which were located in China (Figure 1).

Climate data in China including the current (1970–2000) and future (2050s: 2041–2060; 2070s: 2061–2080) were obtained from the World Climate Database (WorldClim v2.1, https://worldclim.org/data/cmip6/cmip6climate.html, assessed on 5 January 2024) with a spatial resolution 2.5 arcmin (5 km). The global climate model BCC–CSM2–MR (modeling data from the Beijing Climate Center Climate System Model) was employed as future climate, which was driven by different socio-economic assumptions, named as Shared Socio-economic Pathways (SSP) [SSP126 (sustainable development), SSP245 (moderate development), SSP370 (regional development), and SSP585 (normal development)] [28]. As *N. asiatica* pupates in soil, soil characteristics may influence its eclosion and survival [29]. The topsoil data in China were extracted from the Harmonized World Soil Database (HWSD v1.2, https://www.fao.org/soils-portal/data-hub/soil-maps-and-databases/harmonized-world-soil-database-v12/en/, assessed on 5 January 2024) with a spatial resolution of 2.5 arcminutes (5 km). Above all, a total of 36 environmental factors (19 climatic and 17 soil factors) were initially used to construct the MaxEnt model.

In order to avoid overfitting MaxEnt model caused by multicollinearity among environmental factors, Pearson correlation analysis between current environmental factors was performed using ENMTools (https://github.com/danlwarren/ENMTools/, assessed on 24 April 2024) [30]. Then, all the environmental factors were imported into the MaxEnt model (v3.4.1, https://biodiversityinformatics.amnh.org/open_source/maxent/, assessed on 24 April 2024), and factors with zero contribution rates were removed. If two factors had a correlation coefficient larger than 0.8, only the factor with the highest contribution was retained [10]. Finally, 15 environmental factors, including seven climatic and eight soil factors, were retained to construct the model.

### 2.2. MaxEnt Model Optimization and Construction

The reliability of MaxEnt model was closely related to the regularization multiplier (RM) and feature combinations (FCs). The feature combinations contained five basic parameters: linear (L), quadratic (Q), hinge (H), product (P), and threshold (T) along with 31 possible FCs. The Kuenm package (https://github.com/marlonecobos/kuenm, assessed on 24 April 2024) was utilized to calibrate RM and FCs for MaxEnt optimization. The RM was set to 0.1–4 with an interval of 0.1, and then the 31 FCs were employed to test the 40 RMs [31]. A total of 1240 candidate models were generated, and the best models were selected based on following criteria: significant models with omission rates ≤ 5%, and the lowest delta-corrected Akaike information criterion (ΔAICc) values ≤ 2% [32].

The screening distributional records and environmental factors of *N. asiatica* were imported into the optimal MaxEnt model, with 75% of the distribution records as training data and the remaining 25% as testing data. The other parameters were set as follows: “Create responsive curves”, “Do jackknife to measure variable importance”, “Out format logistic”, “Random seed”, “Replicates 10”, “Replicated run type bootstrap”, “Write plot data”, and “Write background predictions” [10].

### 2.3. MaxEnt Model Evaluation

The performance of the optimal MaxEnt prediction was evaluated using the area under the receiver operation characteristic curve (AUC) and true skill statistic (TSS) [32]. The AUC and TSS values ranged from 0 to 1.0 and −1 to 1, respectively, with higher values indicating better model performance. AUC < 0.7 indicates the prediction is extremely poor accuracy, 0.7 < AUC < 0.8 indicates moderate accuracy, 0.8 < AUC < 0.9 indicates good accuracy, 0.9 < AUC < 1 indicates excellent accuracy [33]. The evaluation criteria of TSS were divided into five categories: less than 0.4, fail; 0.4–0.55, fair; 0.55–0.7, good; 0.7–0.85, very good; and 0.85–1.0, excellent [33,34].

### 2.4. Classification and Area Calculation of Suitable Habitats

The maximum training sensitivity plus specificity (MTSPS) was utilized to divide the habitats into suitability [(habitat suitability index (HSI) ≥ MTSPS)] and unsuitability (HSI < MTSPS). The suitable habitats were divided into low suitability (MTSPS–0.4), moderately suitability (0.4–0.6), and highly suitability (0.6–1) [32].

The number of grids occupied by each suitable habitat and the total number of grids (bio9) were calculated using ArcGIS. The area of each habitat in China and the primary goji berry cultivation provinces (Ningxia, Qinghai, Gansu, Xinjiang, and Inner Mongolia) was calculated based on their grid proportion to China’s land area [10,32].

### 2.5. Spatiotemporal Changes in Suitable Habitats

The prediction results were converted into binary files (0 unsuitability, 1 suitability) with a threshold of MTSPS using the SDM Toolbox (v2.5, http://www.sdmtoolbox.org/downloads, assessed on 27 April 2024) in ArcGIS. The “2. Distribution Changes Between Binary SDMs” and “1. Centroid Changes (Lines)” functions in SDM Toolbox were employed to determine the spatiotemporal and centroid changes in suitable habitats.

## 3. Results

### 3.1. Model Optimization and Evaluation

**Figure 2:**
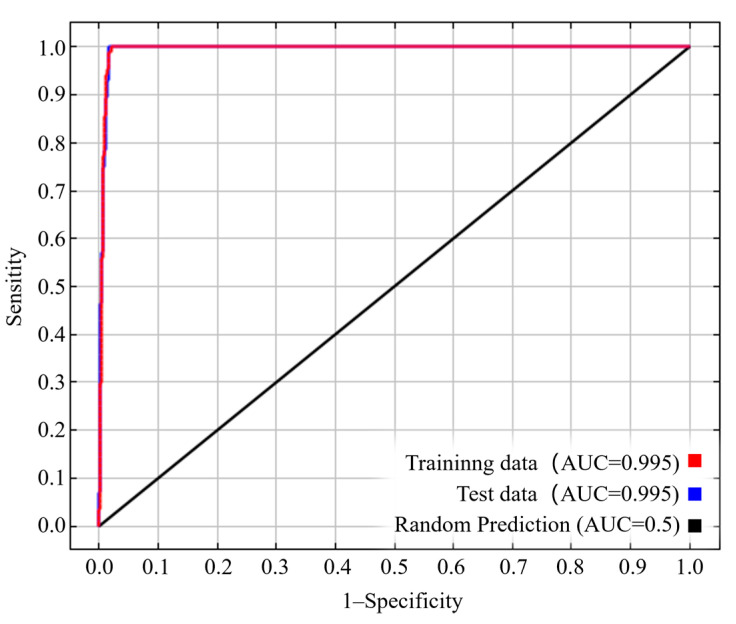
ROC curve and AUC value of *N. asiatica* generated by MaxEnt under current climate conditions.

Based on the results output by Kuenm, when the parameters RM and FC were separately set to 1 and LQPT, ΔAICc was observed to be 0, with an omission rate of 0.036 (Supplementary Figure S1). Therefore, RM = 1 and FC = LQPT were the optimal parameters for MaxEnt. The simulation results showed that the average training AUC and TSS were 0.995 (Figure 2) and 0.979, respectively, indicating the high accuracy and reliability of the optimized MaxEnt model.

### 3.2. Dominant Environmental Factors

As shown in Table 1, the cumulative contribution rates of climate factors were 91.4%, surpassing those (8.6%) of soil factors, indicating that pest distribution was primarily influenced by the climate rather than the soil. The mean temperature of the driest quarter (bio9), the precipitation of the coldest quarter (bio19), and the mean temperature of the coldest quarter (bio11) were the top three dominant environmental factors influencing pest distribution, with a cumulative contribution rate of 77.8% and permutation importance of 66.8%.

Based on the jackknife test using a single environmental factor, bio11, bio9, and bio19 ranked highest in terms of regularized training gain (Supplementary Figure S2), indicating these factors provided more effective information and exerted a stronger impact on pest distribution.

**Figure 3:**
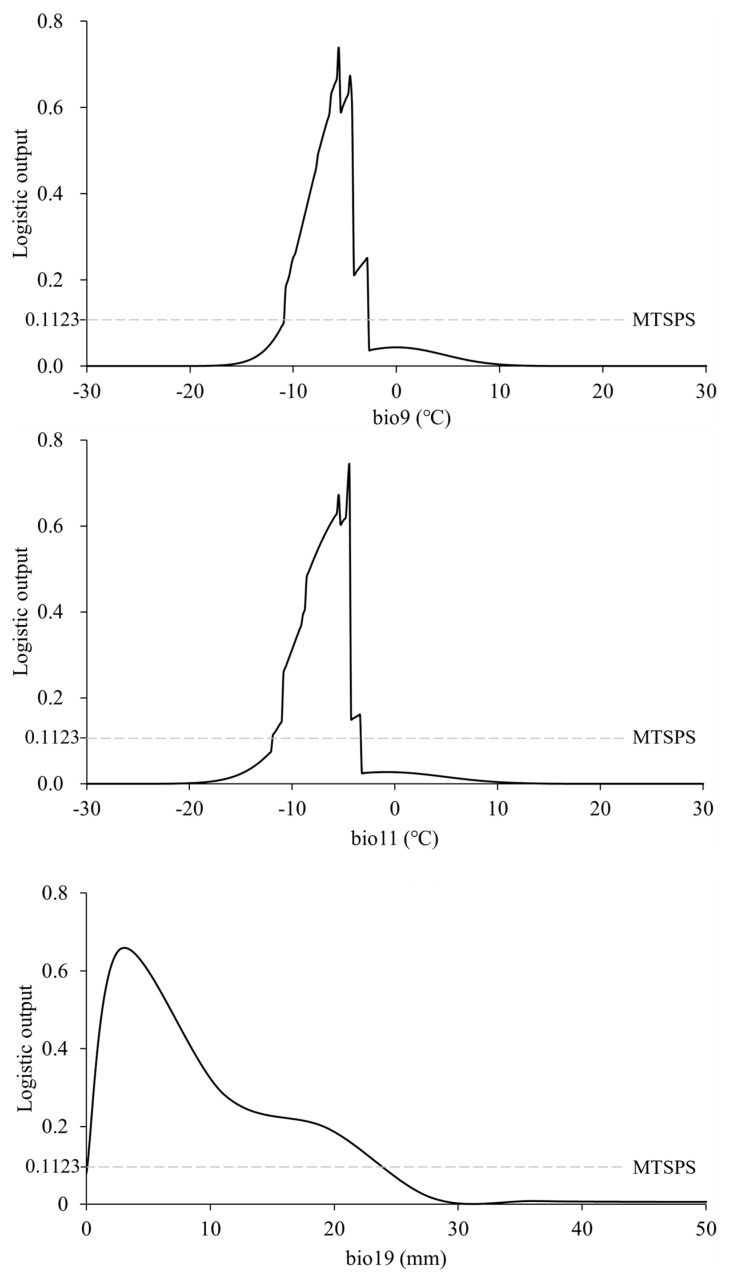
Response curves between the distributional probability and the dominant environmental factors.

Comprehensive jackknife testing and percent contribution analysis indicated that pest distribution under current climatic conditions is predominantly influenced by bio9, bio11, and bio19. According to the response curves between environmental factors and distributional probability, the suitable ranges (distribution probability > MTSPS, MTSPS = 0.1123) of bio9, bio11, and bio19 for the pest were −10.72–−2.82 °C, −11.89–−3.39 °C, and 2.79–19.52 mm (Figure 3), respectively.

### 3.3. Suitable Habitats Under Different Climate Scenarios

#### 3.3.1. Current Suitable Habitats

**Figure 4:**
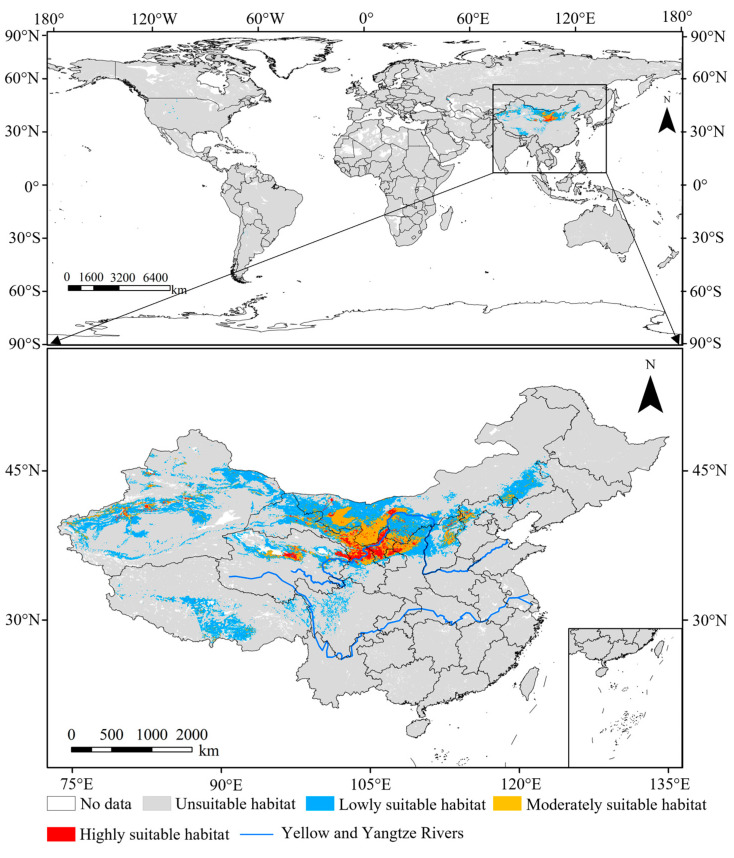
Suitable habitats of *N. asiatica* under current climate conditions [Inspection number: GS (2019) 1822].

As shown in Figure 4, the current suitable habitats of this pest were primarily distributed in China, accounting for 94.49% of those in the world. The area of suitable habitats in China was 141.07 × 10^4^ km^2^, accounting for 14.69% of China’s land area, which was primarily distributed between 27° N–47° N and 73° E–115° E.

**Figure 5:**
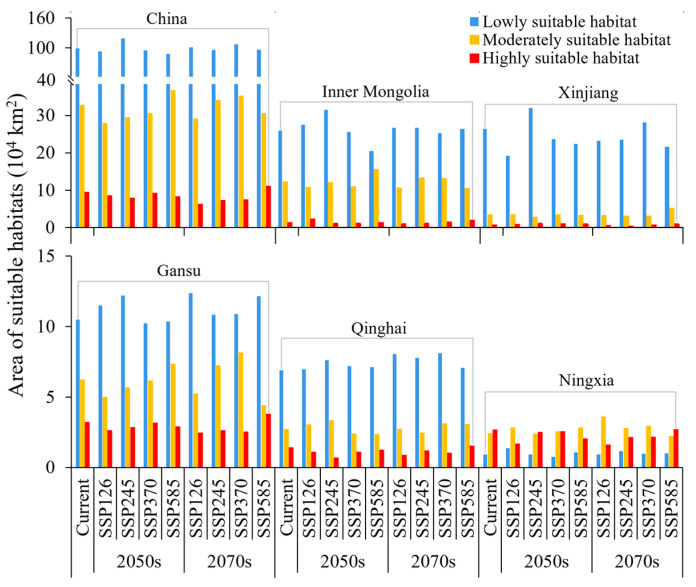
Area of the suitable habitats of *N. asiatica* under current and future climate scenarios.

The moderately suitable habitats (32.67 × 10^4^ km^2^) were primarily situated in the adjacent regions of those with high suitability, making up 23.16% of the suitable habitats in China (Figure 5). The highly suitable habitats (9.51 × 10^4^ km^2^) were concentrated in central Gansu (3.20 × 10^4^ km^2^, 33.65% of those in China), Ningxia (2.67 × 10^4^ km^2^, 28.08%), and eastern and central Qinghai (1.40 × 10^4^ km^2^, 14.72%) Provinces. There were also fragmental distributions of highly suitable habitats in Inner Mongolia (1.21 × 10^4^ km^2^, 12.73%), Xinjiang (0.62 × 10^4^ km^2^, 6.52%), and Shaanxi (0.53 × 10^4^ km^2^, 5.57%) Provinces.

#### 3.3.2. Future Suitable Habitats

The future distribution was highly consistent with the current ones and predominantly located in China (Supplementary Figure S3). The future suitable habitats in China accounted for more than 91% of those in the world.

**Figure 6:**
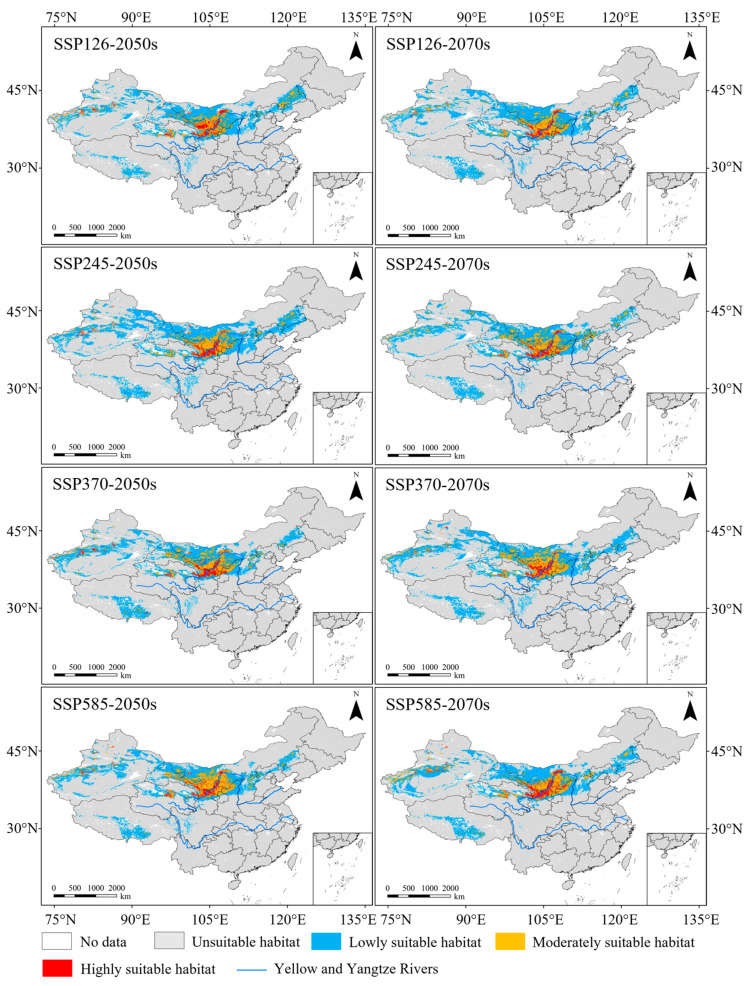
Suitable habitats of *N. asiatica* under different future climate scenarios [Inspection number: GS (2019) 1822].

Different future climate scenarios exerted diverse impacts on the pest distribution (Figure 6). In the 2050s and 2070s, the suitable area reached the maximum under SSP245 (156.57 × 10^4^ km^2^) and SSP370 (149.80 × 10^4^ km^2^), respectively, with increases of 10.99% and 6.19% compared with the current ones. However, the suitable area under the other climate scenarios was all lower than the current ones, decreasing by 2.21–8.41%. The moderately and highly suitable habitats reached the maximum under SSP585–2050s (44.93 × 10^4^ km^2^) and SSP370–2070s (42.62 × 10^4^ km^2^), increasing by 6.51% and 1.05%, respectively. Under the other future climate scenarios, the moderately and highly suitable area decreased by 5.85–13.70% in the 2050s and 1.46–16.32% in the 2070s, respectively (Figure 5).

Among the major goji berry cultivation provinces, Inner Mongolia possessed the largest suitable area for the pest, followed by Xinjiang, Gansu, Qinghai, and Ningxia Provinces (Figure 6). In the 2050s and 2070s, the area of suitable habitats in Xinjiang (36.62 × 10^4^ km^2^) and Inner Mongolia (45.71 × 10^4^ km^2^) reached their maximum under SSP245–2050s, with increases of 18.16% and 13.72%, respectively. In Gansu and Qinghai, the suitable area reached their maximum under SSP370–2070s, increasing by 8.23% and 11.15%, respectively. In Ningxia, the suitable area reached its peak (6.11 × 10^4^ km^2^) under SSP126–2070s, increasing by 1.83%. (Figure 5).

### 3.4. Spatiotemporal and Centroid Changes in the Suitable Habitats of N. asiatica

**Figure 7:**
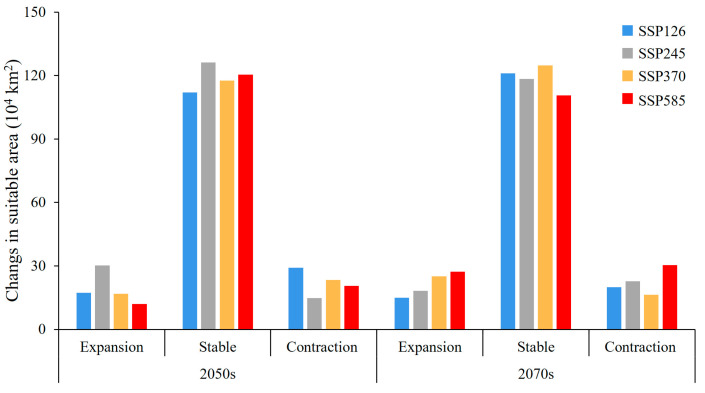
Spatiotemporal changes in the area of suitable habitats under different future climate scenarios compared with the current.

**Figure 8:**
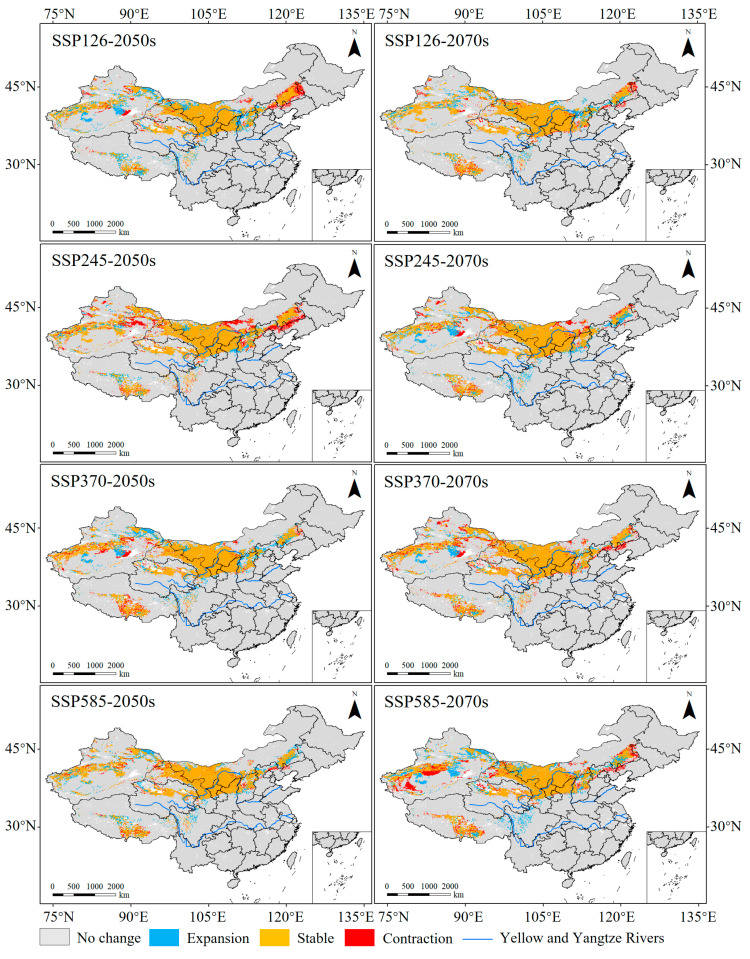
Dynamic changes in suitable habitats of *N. asiatica* under different climate scenarios [Inspection number: GS (2019) 1822].

Compared with the current climatic conditions, 78.44–96.82% of suitable habitats under future climatic scenarios remained unchanged (Figure 7). Except for the other scenarios, the expansion area was all larger than the contraction under SSP245–2050s and SSP370–2070s (Figure 8), and the expanding regions were concentrated in the bordering regions between Liaoning and Inner Mongolia Provinces, northern Hebei, central Inner Mongolia, and eastern Xinjiang Provinces.

**Figure 9:**
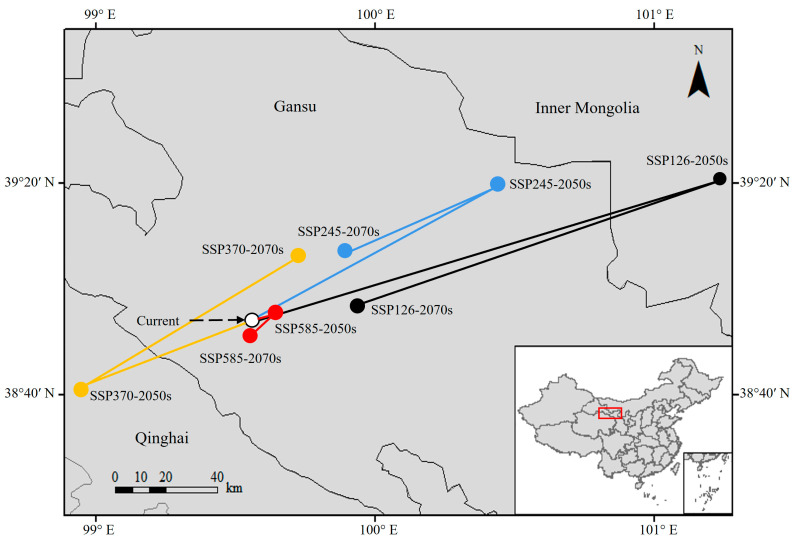
Changes in the centroid of the potential distribution of *N. asiatica* [Inspection number: GS (2019) 1822].

The centroid of the suitable habitats under current climate conditions was located in Zhangye prefecture-level city (99.55° E, 38.91° N), Gansu Province, bordering Qinghai (Figure 9). In the 2050s, the distributional centroids mainly shifted to the northeast of the current ones, with the exception of SSP370, which exhibited a southwestward shift (98.92° E, 38.70° N) covering a distance of approximately 59.20 km. In the 2070s, the changes in centroids shifted towards the southwest of those in the 2050s, except SSP370, which experienced a northeastward shift (99.72° E, 39.10° N).

## 4. Discussion

### 4.1. Dominant Environmental Factors Influencing the Distribution of N. asiatica

Climate including temperature and precipitation plays a crucial role in the distribution of insects [6]. The survival and development of insects are influenced by temperature, which impacts their metabolic processes [35], and precipitation, which affects their water balance within the idiosoma [36]. In this study, we found that the contribution (91.4%) and the permutation (96%) of climate factors were significantly greater than those of soil, indicating that the distribution of *N. asiatica* was primarily influenced by climate rather than soil. This characteristic was reported in many other insects. For instance, the distribution of the Siberian tortoise beetle, *Rhinoncus sibiricus* Faust, was mainly influenced by temperature (26.8% contribution) and precipitation (45%) instead of soil (8.8%) [37]. Our results demonstrated that the mean temperature of the driest quarter (bio9), the mean temperature of the coldest quarter (bio11), and the precipitation of the coldest quarter (bio19) were the dominant environmental factors influencing the geographical distribution of *N. asiatica*.

For insects, winter is the most critical quarter for survival, as they cannot withstand temperatures below their minimum lethal threshold or they will perish [38]. Meanwhile, excessively high winter temperatures will lead to repeated cycles of freezing and thawing in the body fluids of insects, rendering them incapable of survival [39]. We found that the suitable range of mean temperature of the driest quarter and mean temperature of the coldest quarter for *N. asiatica* were −10.72–−2.82 °C and −11.89–−3.39 °C, respectively, indicating that the fruit fly exhibits a preference for temperate climates.

Precipitation in winter also plays a vital role in insect overwintering [6]. The increase in winter precipitation leads to excessive soil moisture, resulting in a reduction in soil oxygen content and subsequently decreasing the survival of overwintering insects [40]. For example, Huang et al. demonstrated a significant decline of 83.33% in the overwintering survival rate of cotton bollworm, *Helicoverpa armigera* Hübner, when the soil moisture reached 60% [41]. Meanwhile, limited winter precipitation exacerbated water evaporation in overwintering insects, thereby exerting a detrimental impact on their survival [6]. For example, the overwintering mortality rate of cabbage maggot, *Hylernya brassicxe* Bouche, eggs in the field reached 90% due to limited snowfall throughout winter [42]. Our results showed that the suitable range of precipitation of the coldest quarter for *N. asiatica* was 2.79–19.52 mm.

### 4.2. Changes in Suitable Habitats of N. asiatica

We found that the suitable habitats of *N. asiatica* were currently distributed in the ranges of 27°–47° N and 73°–115° E, and the moderately and highly suitable habitats were restricted to southwest Inner Mongolia, central Gansu, Ningxia, and Qaidam Basin in Qinghai, which covered the majority of the goji berry cultivation regions in China [13].

Under global climate warming scenarios, the severity of pest infestations is anticipated to escalate, as ambient temperatures progressively approach optimal levels for the development of pests, potentially alleviating thermal constraints on population dynamics [6]. However, our results revealed a reduction in the suitable area of *N. asiatica* under future 2050s and 2070s climate scenarios, ranging from 2.08% to 8.41%, with the exception of SSP 245–2050s and SSP 370–2070s. This difference may be caused by the narrow niche requirements and poor physiological tolerance of the pest. Lehmann et al. [43] indicated that the responses of various pests to climate warming exhibited considerable diversity, with 59% of them expected to mitigate their detrimental impact primarily through reduced physiological performance and contraction in geographic range. Additionally, the warming climate also plays a crucial role in determining the abundance and distribution of pests by influencing the availability of host plants [6]. Wang et al. [9] found that the potentially suitable habitats of goji berry were projected to decrease under future climate scenarios. The fragmentation of suitable habitats for the host plant may lead to a contraction in the geographic distribution range of *N. asiatica* in the future. Similarly, the distribution of the leaf beetle, *Cerotoma trifurcata* Forster, was predicted to be constrained by its host availability limitations [44].

Different pest species have varying requirements for temperature and precipitation, thus resulting in differences in their geographical ranges [45]. It was documented that the distribution ranges of pests are anticipated to migrate to higher latitude zones at a rate of approximately 16.9 km every decade [46]. We found that the geographical centroids of the suitable habitat of *N. asiatica* were mainly shifted to the northeast under climate warming conditions, which was consistent with its host the goji berry. Li et al. demonstrated that the habitat centroids of the *Daodi* goji berry were projected to shift towards the northeast in the future [10]. This phenomenon facilitated the pest in achieving synchronization with its host.

### 4.3. Potential Limitations

In this study, the current and future distributions of *N. asiatica* and the primary environmental factors were identified, which was conducive to the pest monitoring and management. We advise choosing regions free of the pest for goji berry cultivation to eliminate the risk of damage. In regions suitable for pest distribution but currently uninfected, it is crucial to implement proactive monitoring measures to prevent pest spread. For regions moderately to highly suitable for the pest, comprehensive prevention and control strategies should be implemented to reduce damage. However, the distribution of insects is influenced by numerous factors, such as altitude, human activities, natural enemies, as well as their own capacity for adaptation and evolution [47]. However, only climate and soil factors were considered in this study, and pest adaption was considered unchanged when MaxEnt predicted species distribution [48]. Consequently, the predicted suitable habitats may deviate from the actuality. Future research should consider these factors to make a more accurate prediction for *N. asiatica* under climate change conditions.

## 5. Conclusions

In this study, the MaxEnt model with optimized parameters was employed to predict the current and future distributions of *N. asiatica* for the first time, and the dominant environmental factors influencing its distribution were identified. Our results indicated that the primary environmental factors were the mean temperature of the driest quarter, the mean temperature of the coldest quarter, and the precipitation of the coldest quarter. Under current and future (2050s and 2070s) climate scenarios, the suitable habitats were primarily distributed in the northwestern regions of China, and the moderately and highly suitable habitats were concentrated in Ningxia and Gansu Provinces, followed by Qinghai Province. Notably, the suitable area under future climate scenarios was all lower than the current ones, except for SSP245–2050s and SSP370–2070s. The centroids of suitable habitats were mainly shifted to the northeast in the 2050s and 2070s, except for SSP370–2050s and SSP585–2070s. Our results provide guidance for the monitoring and management of *N. asiatica*, as well as for selecting cultivation sites that are free from pest damage.

## Figures and Tables

**Table 1:** Percent contribution and permutation importance of the dominant environmental factors.

Factor	Description	Percent Contribution (%)	Permutation Importance (%)
bio9	Mean temperature of driest quarter (°C)	31.8	51.9
bio19	Precipitation of coldest quarter (mm)	27.6	2.5
bio11	Mean temperature of coldest quarter (°C)	18.4	12.4
bio12	Annual precipitation (mm)	6.3	2.5
bio2	Mean diurnal range (°C)	5.4	0.2
t_bs	Topsoil base saturation (%)	3.4	1.3
t_gravel	Topsoil gravel content (%)	3.3	1
bio15	Precipitation seasonality (mm)	1.7	0.5
t_silt	Topsoil silt fraction (%)	0.9	0.8
t_caco3	Topsoil calcium carbonate (%)	0.8	0.7
bio14	Precipitation of driest month (mm)	0.2	26
t_caso4	Topsoil gypsum (%)	0.2	0.2
t_cec_soil	Topsoil cec (soil) (cmol/kg)	0	0.1
t_cec_clay	Topsoil cec (clay) (cmol/kg)	0	0
t_clay	Topsoil clay fraction (%)	0	0

## Data Availability

Data is contained within the article.

## Supplementary Materials

The following supporting information can be downloaded at: https://www.mdpi.com/article/10.3390/insects15080558/s1, Table S1: Distribution records of *N. asiatica*; Figure S1: The results of model optimization; Figure S2: Jackknife test of the importance of environment factors of *N. asiatica*; Figure S3: Global potential distribution of *N. asiatica* under different future climate scenarios.
