# Supplementary figures and images for: Predicting the Distribution of Neoceratitis asiatica (Diptera: Tephritidae), a Primary Pest of Goji Berry in China, under Climate Change
Source: Insects. 2024 Jul 23;15(8):558. doi: 10.3390/insects15080558 (PMC11355054; doi:10.3390/insects15080558)

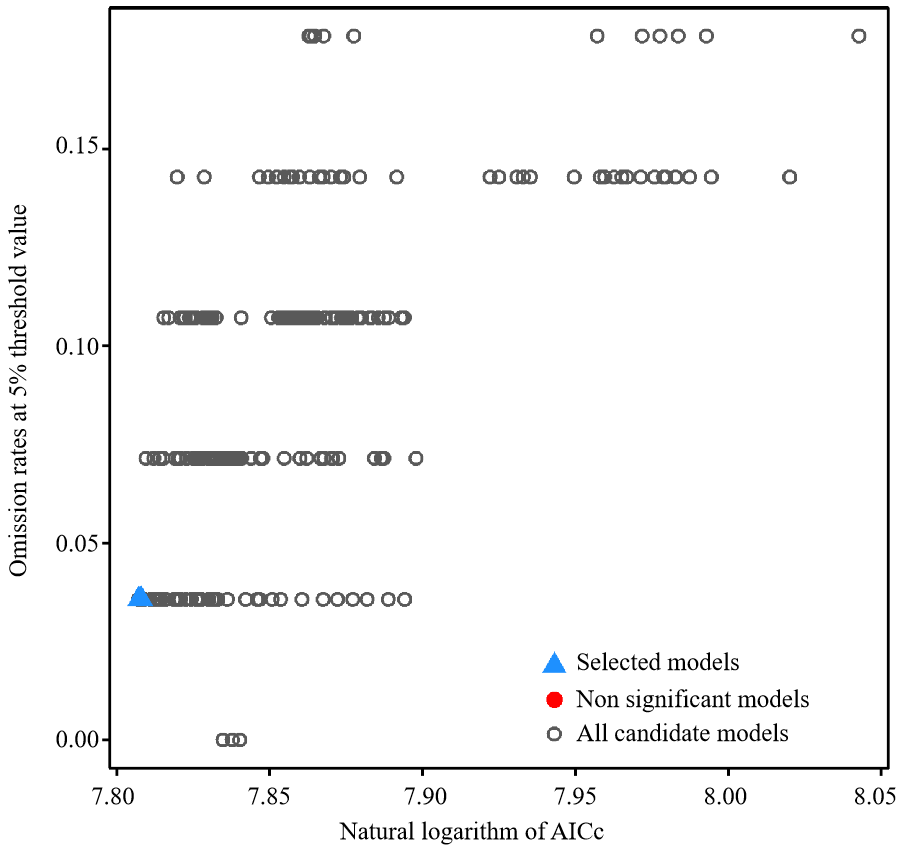

Supplement: Supplementary file 1 [file insects-15-00558-s001.zip › Figure S1 The results of model optimization.tif]

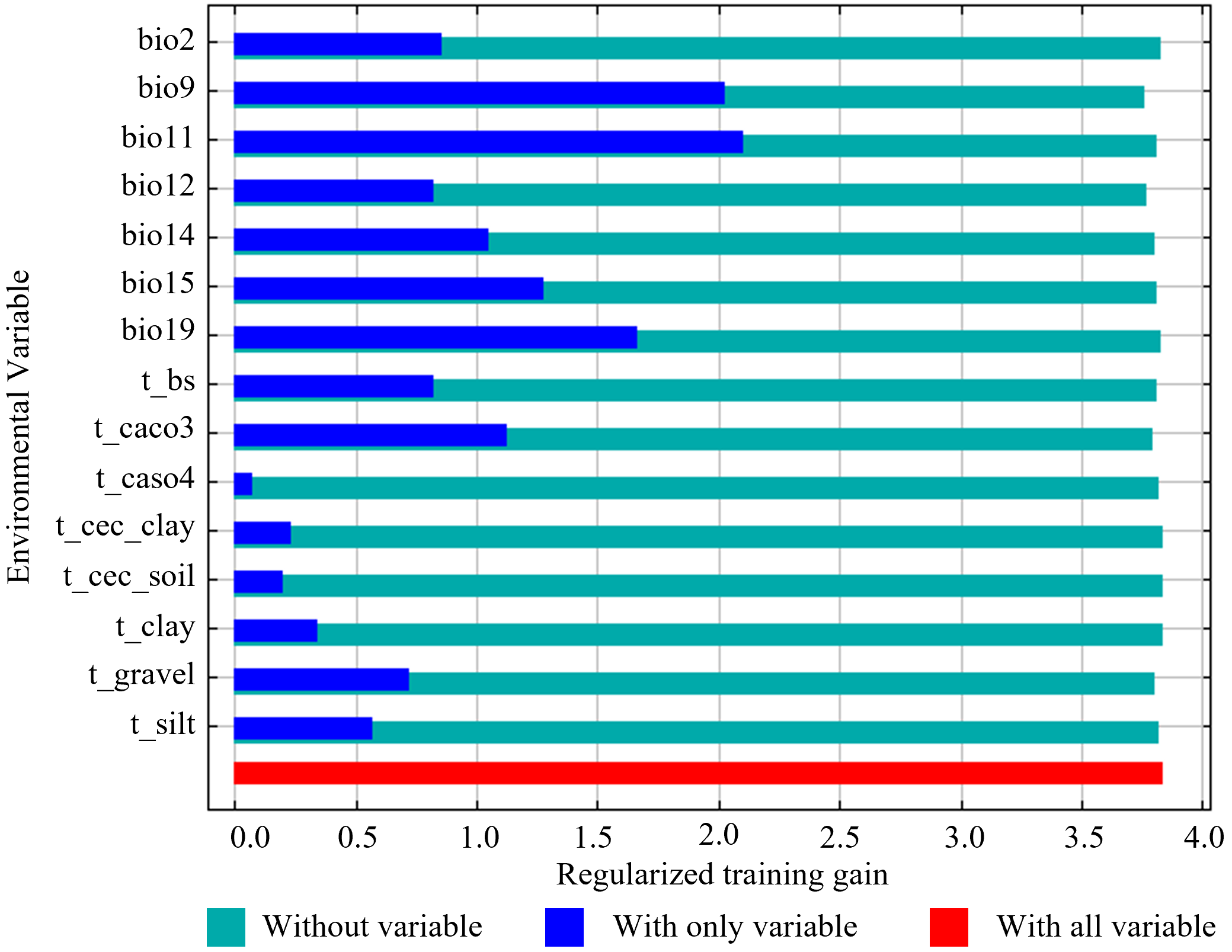

Supplement: Supplementary file 1 [file insects-15-00558-s001.zip › Figure S2 Jackknife test of the importance of environment factors of N. asiatica.tif]

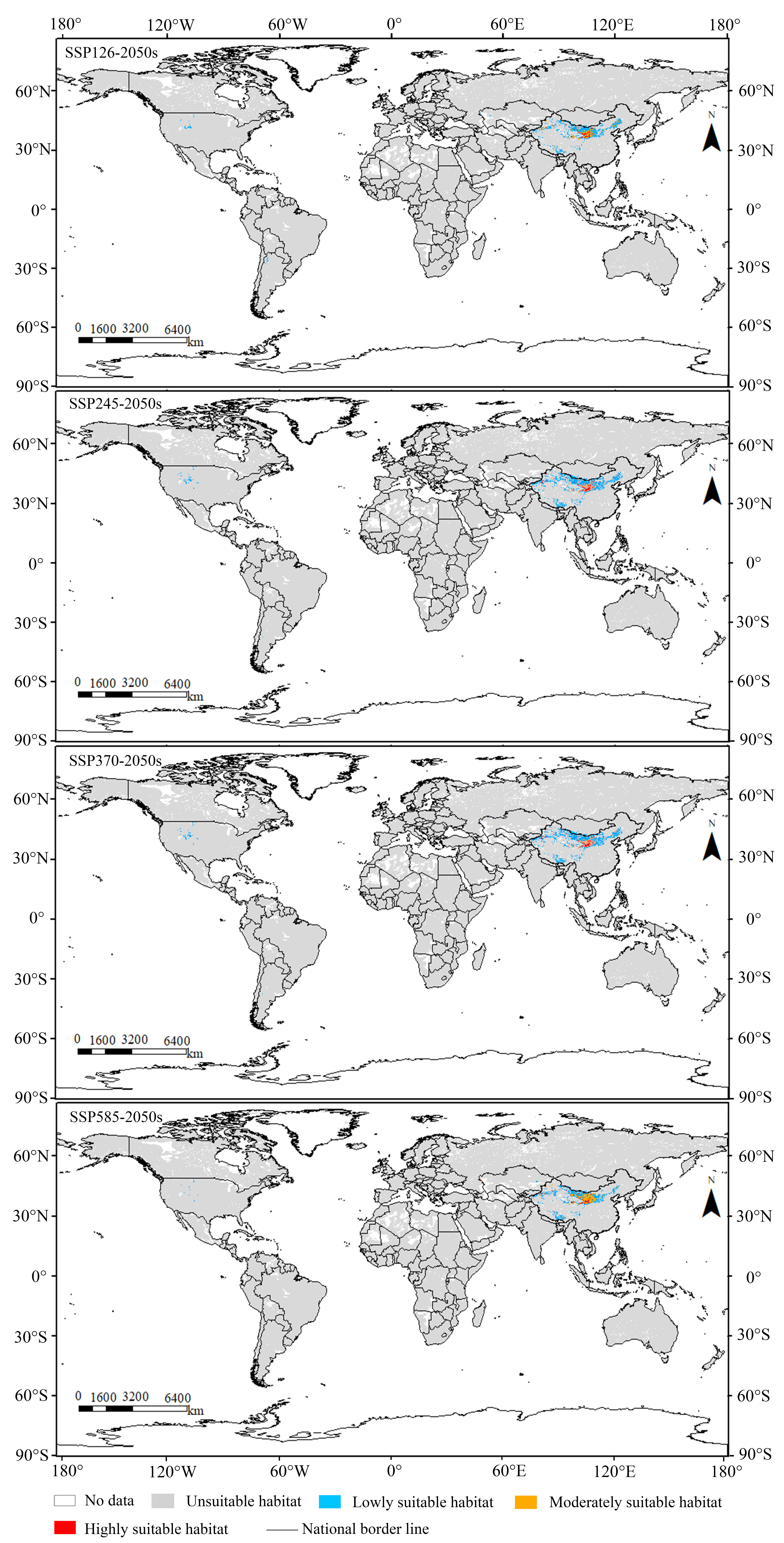

Supplement: Supplementary file 1 [file insects-15-00558-s001.zip › Figure S3 Global potential distribution of N. asiatica under different future climate scenarios (1).tif]

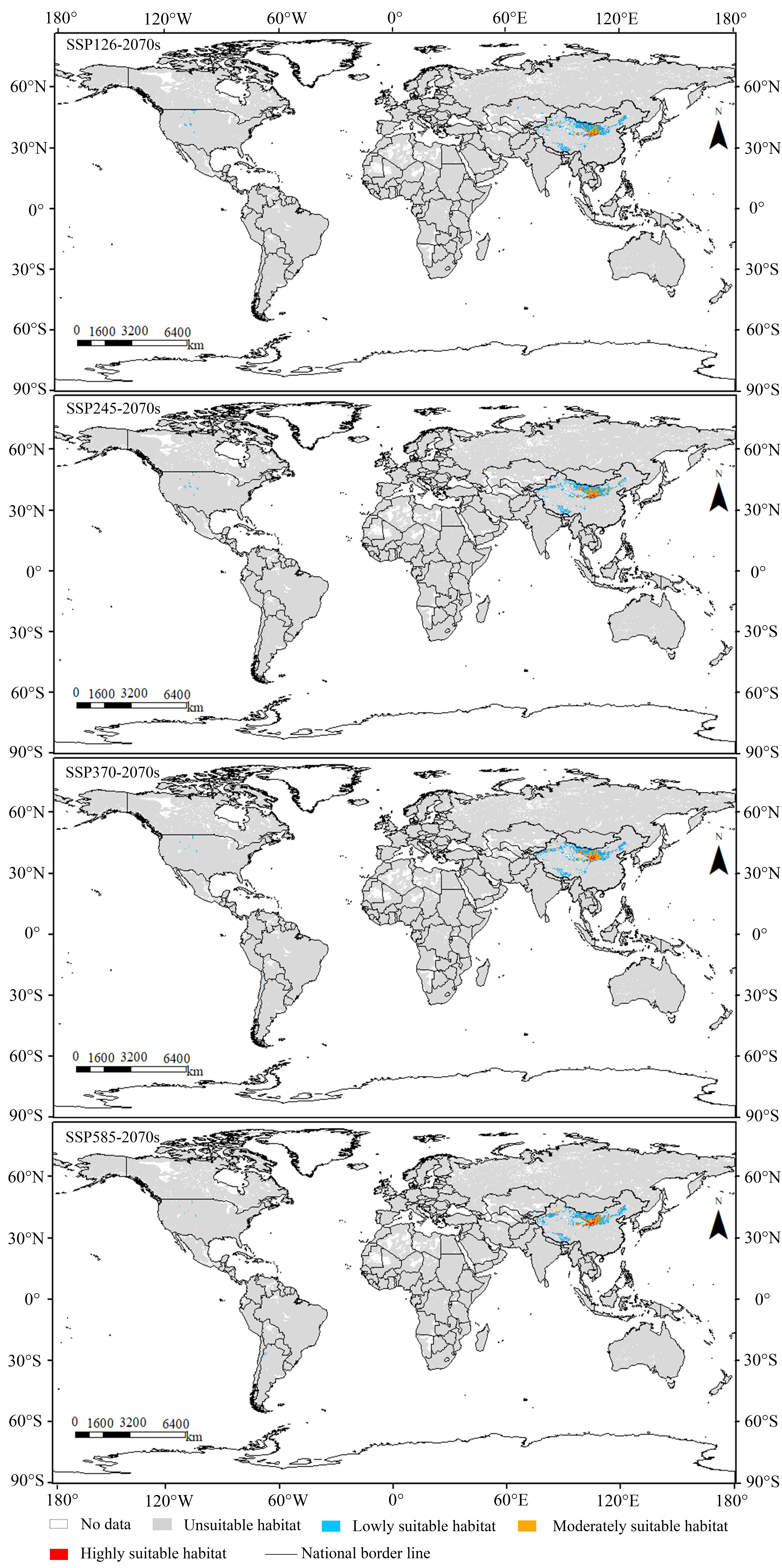

Supplement: Supplementary file 1 [file insects-15-00558-s001.zip › Figure S3 Global potential distribution of N. asiatica under different future climate scenarios (2).tif]
